# A Protective Role for NKG2D–H60a Interaction via Homotypic T Cell Contact in Nonobese Diabetic Autoimmune Diabetes Pathogenesis

**DOI:** 10.4049/immunohorizons.1700011

**Published:** 2017-11-07

**Authors:** Andrew P. Trembath, Neekun Sharma, Saravanan Raju, Bojan Polić, Mary A. Markiewicz

**Affiliations:** * Department of Microbiology, Molecular Genetics and Immunology, University of Kansas Medical Center, Kansas City, KS 66160; † Department of Pathology and Immunology, Washington University School of Medicine, St. Louis, MO 63110; ‡ Department of Histology and Embryology, Medical Faculty, University of Rijeka, 51000 Rijeka, Croatia

## Abstract

The NK group 2 member D (NKG2D) immune receptor is implicated in both human and mouse autoimmune diabetes. However, the significance of NKG2D in diabetes pathogenesis has been unclear due to conflicting reports as to the importance of this receptor in the NOD mouse model. In this study we demonstrate that NKG2D expression affects NOD diabetes development by at least two previously undescribed, and opposing, mechanisms. First, we demonstrate that the NKG2D ligand H60a is induced on activated NOD T cells, and that NKG2D–H60a interaction during CD8^+^ T cell differentiation into CTLs generally decreases the subsequent CTL effector cytokine response. This corresponds to an increase in diabetes development in NKG2D-deficient compared with wild-type NOD mice under microbiota-depleted conditions. Second, we demonstrate that NKG2D promotes NOD diabetes development through interaction with the microbiota. Together these findings reveal a previously undescribed role for NKG2D ligand expression by activated T cells in CTL development. Further, they demonstrate that NKG2D has both diabetogenic and antidiabetogenic roles in NOD diabetes development.

## INTRODUCTION

Type 1 diabetes is an autoimmune disease occurring with increasing frequency worldwide (1). In type 1 diabetes the insulin-producing β cells in pancreatic islets are destroyed by the immune system, resulting in a lifelong dependence on supplemental insulin therapy and reduced life expectancy (2). The cause of type 1 diabetes remains unknown; however, it is clear that many factors contribute to disease development. T cells are believed to play a significant role in type 1 diabetes development, with CD8^+^ T cells being critical to β cell destruction (3–6). Mouse studies implicate the NK group 2 member D (NKG2D) immune receptor in this diabetogenic CD8^+^ response (7–9). Further, genetic linkage studies suggest an association between polymorphismin the gene encoding one of the human NKG2D ligands, MHC class I chain–related A (MICA), and type 1 diabetes (7, 8, 10).

NKG2D, encoded by the gene *Klrk1*, is expressed on all human and mouse NK cells, all human CD8^+^ T cells, activated (but not naive) mouse CD8^+^ T cells, γδ T cells, NKT cells, and rare CD4^+^ T cells in both human and mouse (11–17). NKG2D binds to a number of ligands that are not normally expressed by most healthy tissues, but rather are induced by cellular stress (18). In humans, the NKG2D ligands are MICA, MICB, and the retinoic acid early transcript 1 family members (also known as the UL16-binding proteins, or ULBP1–6) (11, 19–21). The mouse NKG2D ligands are murine ULBP-like transcript 1, the RAE1 protein family (RAE1α-ε), and H60 proteins (H60a–c) (22–26). All ligands induce similar signaling through NKG2D, which in NK cells triggers granule release and cytokine production (27). The function of NKG2D engagement in CD8^+^ T cells is less well understood, with both costimulatory as well as TCR-independent functions, for NKG2D engagement on T cells described (8, 11, 20, 21, 28–31).

In a previous study with the NOD mouse model, expression of RAE1 was observed in the pancreatic islets, and Ab blockade of NKG2D was found to inhibit CD8^+^ T cell infiltration and prevent diabetes development (7). We subsequently showed that transgenic expression of RAE1ε by pancreatic β cells in nonautoimmune C57BL/6 mice caused recruitment of activated CD8^+^ T cells to pancreatic islets (8). This suggested a mechanism by which NKG2D ligand expression in the islets enhances diabetes development in NOD mice. However, RAE1 gene expression was not detected at any time during diabetes development in NOD pancreatic islets in two other studies (32, 33). Additionally, diabetes development in NOD mice genetically deficient in NKG2D was reported to be similar to that of wild-type NOD mice (34). These conflicting results suggest that NKG2D may play a more complex role in autoimmune diabetes development than originally proposed, affecting disease development through multiple mechanisms.

In the current study, we compared effector CTL responses and diabetes development between NOD mice genetically deficient in NKG2D expression (*Klrk1*^−/−^) and wild-type NOD mice. In contrast to what was previously reported (7), we detected the NKG2D ligand H60a, rather than RAE1, in the pancreatic islets of NOD mice. This expression was not on islet cells, but on infiltrating T cells. We also found that both H60a and NKG2D were expressed on NOD CD8^+^ T cells following activation, and that NKG2D–H60a interaction via homotypic CD8^+^ T cell contact during NOD CTL differentiation decreased effector cytokine production by the CTL in vitro. This corresponded with increased effector cytokine production by *Klrk1*^−/−^ NOD CTL in vivo and increased diabetes development in microbiota-depleted *Klrk1*^−/−^ NOD mice. By contrast, microbiota-replete *Klrk1*^−/−^ mice had reduced diabetes development compared with wild-type mice, showing a separate effect resulting from interactions between NKG2D and the microbiota. Taken together, these results demonstrate that NKG2D influences NOD diabetes development by at least two separate, opposing mechanisms; NKG2D–ligand interaction during CTL generation dampens the diabetogenic CTL response, whereas NKG2D signaling induced by interaction with the microbiota promotes diabetogenesis.

## MATERIALS AND METHODS

### Mice

NOD mice were purchased from the Jackson Laboratory. *Klrk1*^−/−^ mice on the C57BL/6 background have been previously described (35). The *Klrk1*^−/−^ allele was moved from the C57BL/6 to the NOD genetic background using the speed congenics service of the Washington University School of Medicine to generate *Klrk1*^−/−^ NOD mice. By single nucleotide polymorphism analysis (performed by DartMouse), these mice are 98% NOD, with the only observed genomic region containing a continuous interval of non–NOD-like single nucleotide polymorphisms found at the expected site of the *Klrk1* knockout allele. Experiments were performed with *Klrk1*^+/+^ (wild-type) and *Klrk1*^−/−^ NOD littermates from *Klrk1*^+/−^ NOD interbreeding. Mice were housed under specific pathogen-free (SPF) conditions in the Washington University School of Medicine or University of Kansas Medical Center animal facilities in accordance with institutional guidelines.

### Abs

Unconjugated anti-CD28 (Clone 37.51), anti-CD3ε (Clone 2C11), anti–mCD8-APC, anti–mCD4-BV786, anti–CD3ε-PE-Cy7, anti-mCD107 (LAMP1)-PE, anti–DX5-FITC, and anti-mCD45 were purchased from BD Biosciences. Anti–H60-PE, anti–mNKG2D-PE, rat IgG2a-PE, and rat IgG2b-PE were purchased from R&D Systems.

### Pancreatic islet isolation

Islets were purified with a Ficoll-Paque gradient by published methods (36). Pancreata were minced and digested with collagenase IV (20 mg/ml) (Sigma-Aldrich). The digested pancreata were spun through a Ficoll-Paque gradient and the islets harvested.

### RT-PCR

Single-cell suspensions were generated from whole pancreas, purified islets, or from cells separated into CD45^+^ and CD45^−^ fractions via magnetic bead separation (Invitrogen). Total RNA was isolated with Trizol (Invitrogen) followed by the RNeasy Mini Kit (Qiagen) according to the manufacturers’ protocols. Reverse transcription was performed with the SABiosciences RT^2^ First Strand Kit (QIAGEN). Real-time quantitative PCR was performed using the Power SYBR Green PCR Master Mix (Applied Biosystems) and an ABI 7500 (Applied Biosystems) instrument, according to the manufacturers’ instructions. Transcript levels were normalized to 18S. The sequence of the primers used were: RAE1 (all isoforms): forward 5′-CCACCTGGGAATTCAACA-3′ TC; reverse 5′-TGA TCT TGG CTT TTC CTT GG-3′; H60a: forward 5′-TGC CTG ATT CTG AGC CTT TTC-3′ A; reverse 5′-ATT CAC TGA GCA CTG TCC ATG TAG-3′ AT; 18S: forward 5′-CCGCAGCTAGGAATAATGGAA-3′; reverse 5′-CGAACCTCC GAC TTT CGT TCT-3′.

### In vitro CTL generation

All cells were grown in IMDM (Cellgro) supplemented with 10% defined FBS (HyClone), penicillin-streptomycin-glutamine (Life Technologies), and β-mercaptoethanol (Sigma-Aldrich). For activation of total splenocytes, spleens were harvested from 6 to 8 wk old SPF-housed NOD mice, passed through a 40 µm cell strainer, and plated in a six-well dish at 1.5 × 10^7^ cells per well in 3 ml media and 1 µg/ml of plate-bound anti-CD3ε (2C11). For purified CD8^+^ T cells, cells were harvested from spleens and lymph nodes of 6–8 wk old NOD mice, and enriched by negative selection using magnetic beads (BD Biosciences) according to the manufacturer’s protocol. CD8^+^ T cells were then plated at 3–5 × 10^6^ cells per well in a six-well dish with 1 µg/ml plate-bound anti-C3ε (2C11) and soluble anti-CD28 (37.51). The cells were allowed to expand for 5 d and split as needed.

### Cytokine analysis

#### In vitro

CTL were harvested and plated at 1 × 10^5^ cells per well in 100 µl of fresh media in 96-well plates precoated with anti-CD3 (2C11) at the stated concentrations. Cells were incubated at 37°C for 20 h. Supernatant was then collected and cytokines were analyzed using the Mouse Inflammation Cytometric Bead Array kit (BD Biosciences) and an LSR II (BD Biosciences). Data were quantified using FCAP array software (BD Biosciences).

#### In vivo

Total CD8^+^ T cells were purified from spleens and lymph nodes of 6–8wk old *Klrk1*^−/−^ or wild-type NOD mice by negative selection using magnetic beads (BD Biosciences) according to the manufacturer’s protocol. Isolated *Klrk1*^−/−^ and wild-type CD8^+^ T cells were then labeled with eFluor 670 (eBioscience) and CFSE (Thermo Fisher Scientific), respectively. The labeled cells were then mixed 1:1 and adoptively transferred via retro orbital injection into 6–8 wk old wild-type NOD recipient mice (Supplemental Fig. 2A). Total splenocytes were harvested after 7 d, incubated for 4–6 h in complete media with GolgiPlug protein transport inhibitor (BD Biosciences), followed by fixation and permeabilization using BD Cytofix/Cytoperm (BD Biosciences) according to the manufacturer’s directions. Cells were then stained overnight at 4°C with anti-mouse TNF-α (BD Pharmingen), anti-mouse IFN-γ (BD Pharmingen), and anti-mouse CD8 (BioLegend), and analyzed by flow cytometry. Transferred CD8^+^ T cells were analyzed by gating on live lymphocytes based on forward and side scatter, CD8^+^ cells, then either eFluor 670^+^ or CFSE^+^ cells.

### Lamp-1 granule release analysis

CTL were plated at 1 × 10^5^ in 100 µl fresh media in 96-well plates precoated with anti-CD3ε (2C11) at the stated concentrations. Before plating, PE-conjugated anti-CD107a was added to the cells at 4 µl/ml. Cells were incubated at 37°C for 3 h before being washed, fixed in 2% paraformaldehyde, and assessed for LAMP1 staining by flow cytometry.

### Antibiotic treatment of mice

A solution of 0.5 g/l vancomycin (Sigma-Aldrich), 1 g/l neomycin (Sigma-Aldrich), 1 g/l ampicillin (Sigma-Aldrich), and 1 g/l metronidazole (Sigma-Aldrich) was made in grape KoolAid. This solution was mixed with Dietgel Boost (ClearH_2_O) and crushed food pellets.

*Klrk1*^−/−^ and wild-type NOD littermates were exclusively fed this antibiotic mixture beginning at weaning until euthanasia. Sequencing of the 16S rRNA genes present in feces confirmed that the Firmicutes and Bacteroidetes populations, the two predominant bacteria phyla present in the intestine, were reduced 94–99% within 4 wk of antibiotic treatment.

### Diabetes determination

In all experiments the age of diabetes development was defined as the age at which the first of two consecutive blood glucose measurements ≥250 mg/dl was obtained.

### Statistical analysis

Data were analyzed using a two-tailed unpaired Mann–Whitney *U* test, one-tailed Wilcoxon test, or two-way ANOVA as described in the figure legends. All statistical analyses were performed using GraphPad Prism.

## RESULTS

### The NKG2D ligand H60a is expressed on infiltrating T cells within the pancreas of NOD mice

**FIGURE 1:**
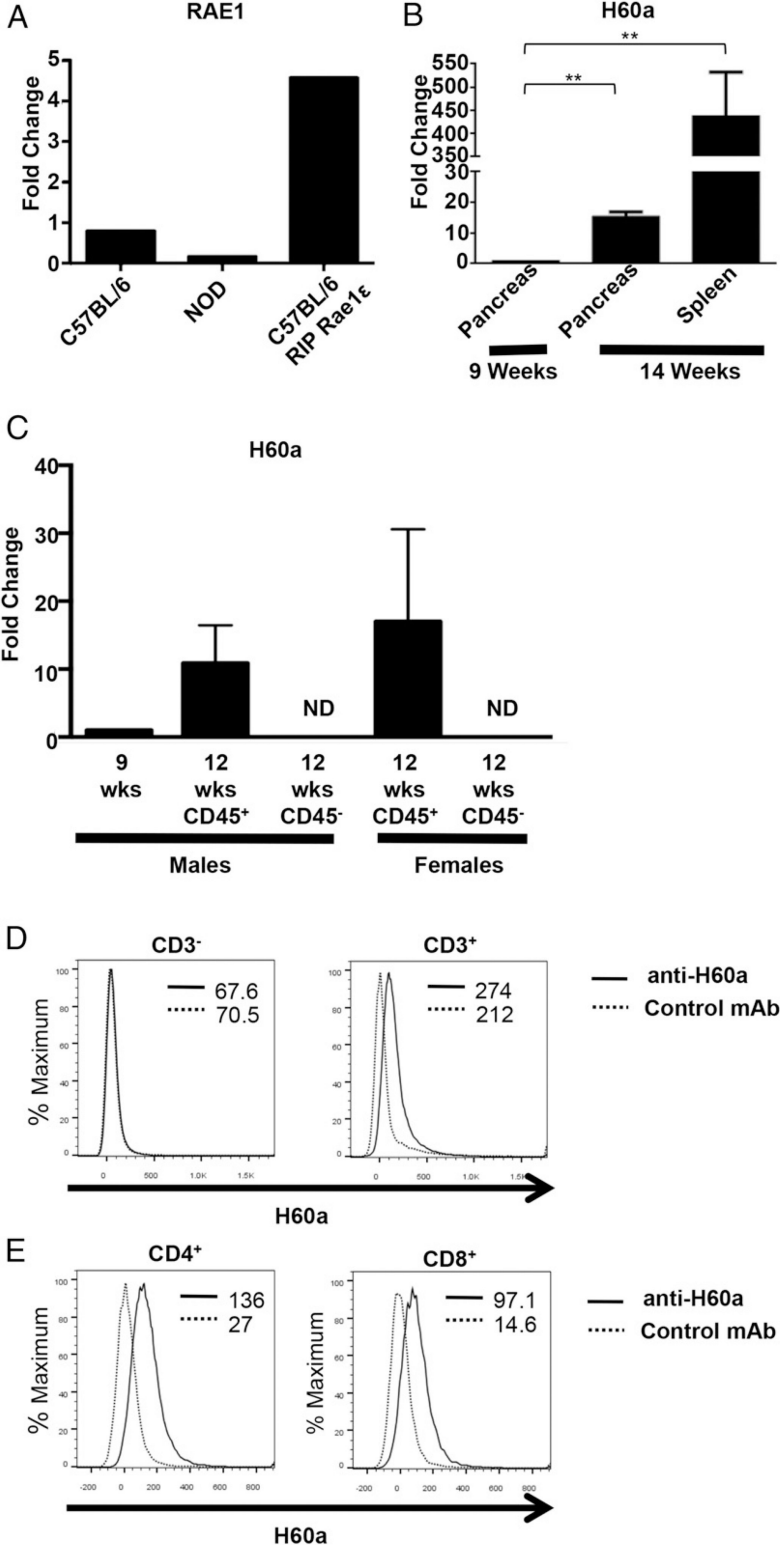
T cells within the pancreas of NOD mice express the NKG2D ligand H60a (**A**) Relative expression of RAE1 mRNA levels in isolated pancreatic islets from 20 wk old C57BL/6, NOD, or RIP-RAE1ε (8) mice. (**B**) Relative expression of H60a mRNA in the pancreas and spleen of 9 (expression set as 1-fold) and 14 wk old NOD mice (mean ± STD). (**C**) Relative expression of H60a mRNA in CD45^+^ and CD45^−^ cells purified from isolated NOD pancreatic islets (mean ± STD). The housekeeping gene 18S was used in all experiments. (**D**) H60a expression on CD3^−^ and CD3^+^ cells within the pancreas of a 12 wk old NOD mouse. (**E**) H60a expression on CD4^+^ and CD8^+^ T cells within the pancreas of a 12 wk old NOD mouse. The mean fluorescence intensity of staining is shown. Data are representative of at least three independent experiments. **p ≤ 0.01 in two-tailed unpaired Mann–Whitney *U* test. ND, not detected.

We set out to better characterize the role of NKG2D and its ligands in NOD diabetes. We first examined wild-type NOD pancreatic islets for NKG2D ligand expression. A previous report described the expression of RAE1 family members in the pancreatic β-islet cells of NOD mice, and suggested that this expression targeted β-islet cells for autoimmune destruction (7). Therefore, we first looked for RAE1 mRNA expression in the pancreatic islets of our NOD mice. We used RIP-RAE1ε mice, which constitutively express RAE1ε in the pancreatic islets (8), as a positive control. However, we were unable to detect RAE1 mRNA in the pancreatic islets of wild-type NOD mice in our colony (Fig. 1A). We next looked for expression of the other NKG2D ligand expressed in NOD mice, H60a. We found significant expression of H60a mRNA in both the pancreas and spleen of NOD mice by 12 wk of age (Fig. 1B, 1C).

To determine whether H60a was expressed within pancreatic islets, as well as which cells expressed H60a, we compared H60a mRNA levels between immune (CD45^+^) and nonimmune (CD45^−^) cells present within isolated islets from 12 wk old mice. These analyses revealed H60a mRNA was expressed in the CD45^+^ immune cells, but not the CD45^−^ islet cells (Fig. 1C). Finally, we determined by flow cytometric analysis that the major cells expressing H60a were pancreas-infiltrating T cells (Figs. 1D, 1E).

### NOD CD8^+^ T cells express NKG2D and H60a upon activation

**FIGURE 2:**
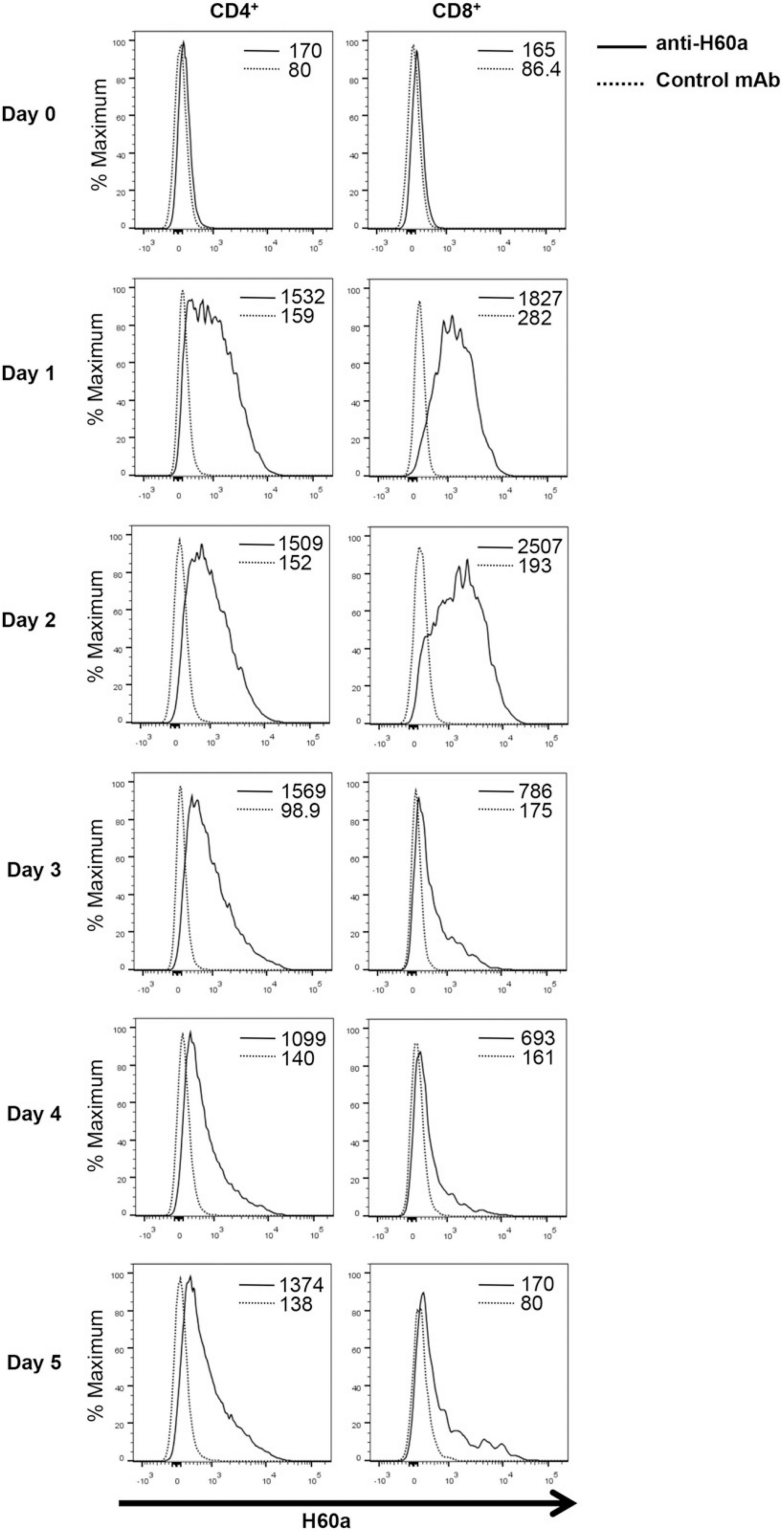
NOD T cells express H60a upon activation H60a expression on CD4^+^ and CD8^+^ T cells following activation of NOD splenocytes with anti-CD3ε Ab. The mean fluorescence intensity of staining is shown. H60a expression was assessed on CD4^+^ and CD8^+^ gated live cells. Data are representative of at least four independent experiments.

We next set out to determine the role of H60a expression by NOD T cells. We first characterized the expression of H60a on splenic T cells in young (6–8 wk old) NOD mice. We observed low but detectable H60a expression on freshly isolated splenic T cells (Fig. 2). We tested whether activation would increase this expression by stimulating NOD splenocytes in vitro with anti-CD3 Ab. H60a expression increased substantially on both CD4^+^ and CD8^+^ T cells following activation and remained elevated through day 5 of culture (Fig. 2).

**FIGURE 3:**
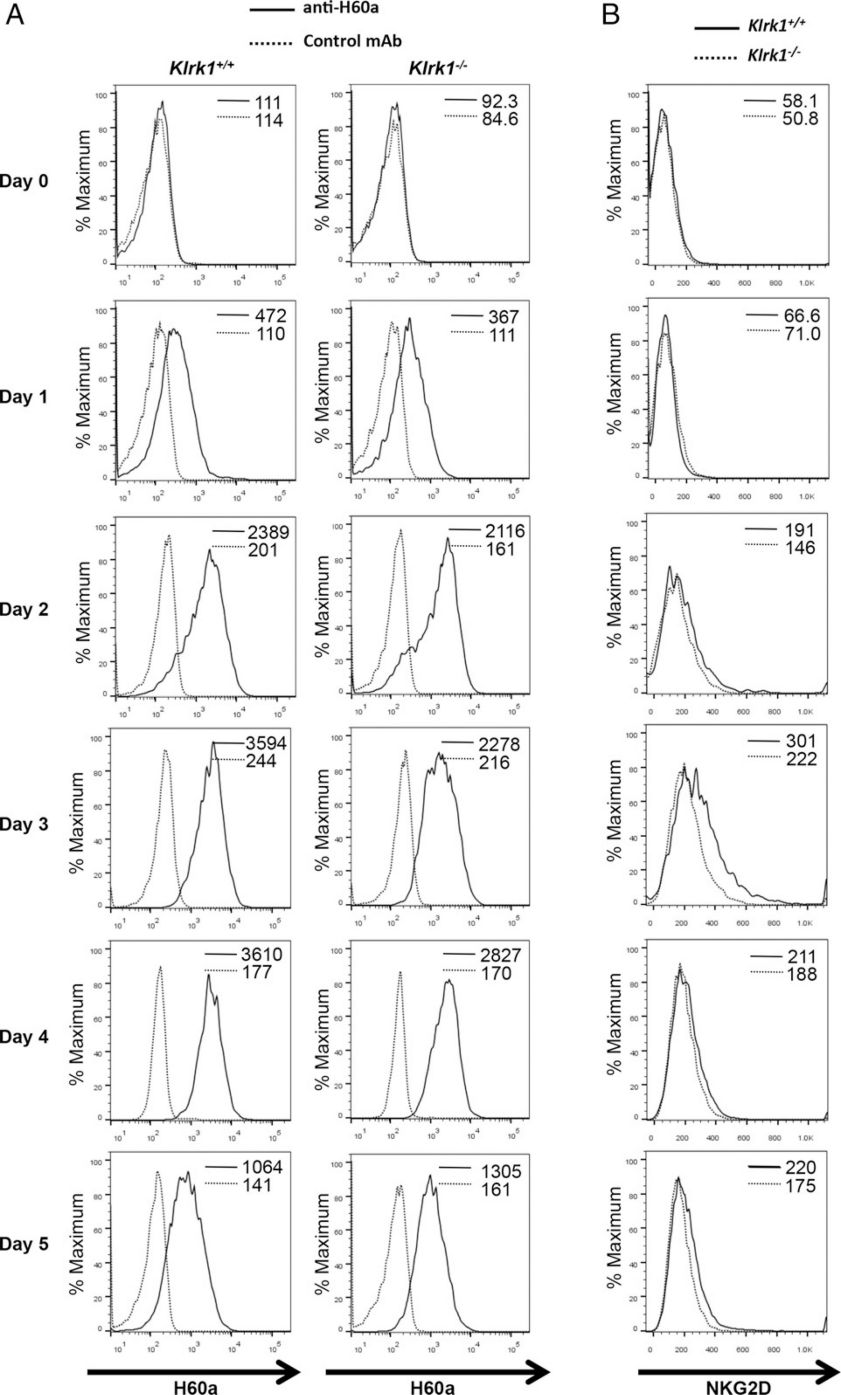
NOD CD8^+^ T cells express both H60a and NKG2D upon activation Expression of (**A**) H60a and (**B**) NKG2D on CD8^+^ T cells purified from 6 to 8 wk old *Klrk1*^−/−^ and wild-type NOD mice activated with anti-CD3ε and anti-CD28 Abs. Mean fluorescence intensity of staining is shown. Data are representative of at least four independent experiments.

In addition to H60a, activated NOD CD8^+^ T cells express NKG2D (7). Therefore, we determined the time course of both H60a and NKG2D expression after initial TCR activation of NOD CD8^+^ T cells in vitro. CD8^+^ T cells were purified from the spleens and lymph nodes of 6–8 wk old *Klrk1*^−/−^ and wild-type NOD mice. NKG2D and H60a expression was assessed at the time of cell isolation and daily following activation with anti-CD3 and anti-CD28 Abs. Similar to what we observed with whole splenocytes (Fig. 2), a low level of H60a was detected directly ex vivo, increased after TCR activation, and continued through day 5 (Fig. 3A). This expression was unaffected by *Klrk1* (Fig. 3A). In wild-type mice, NKG2D became detectable on day 2 and persisted through day 5 (Fig. 3B).

### NKG2D expression alters NOD CTL effector cytokine responses in vitro and in vivo

NKG2D engagement by ligands expressed on target cells enhances CTL effector function induced by TCR engagement by Ag expressed on the same target cells (11, 28). Given our finding that NOD CTL coexpress NKG2D and the NKG2D ligand H60a, we hypothesized NKG2D–H60a interaction via homotypic CD8^+^ T cell contact could similarly alter NOD CTL effector function. To test this, we activated purified *Klrk1*^−/−^ and wild-type NOD CD8^+^ T cells with anti-CD3 and anti-CD28 Abs in vitro and allowed them to differentiate for 5 d. Cytokine production and lytic granule release by the CTL were then assessed after restimulation with various concentrations of plate-bound anti-CD3 Ab to mimic target cell recognition.

**FIGURE 4:**
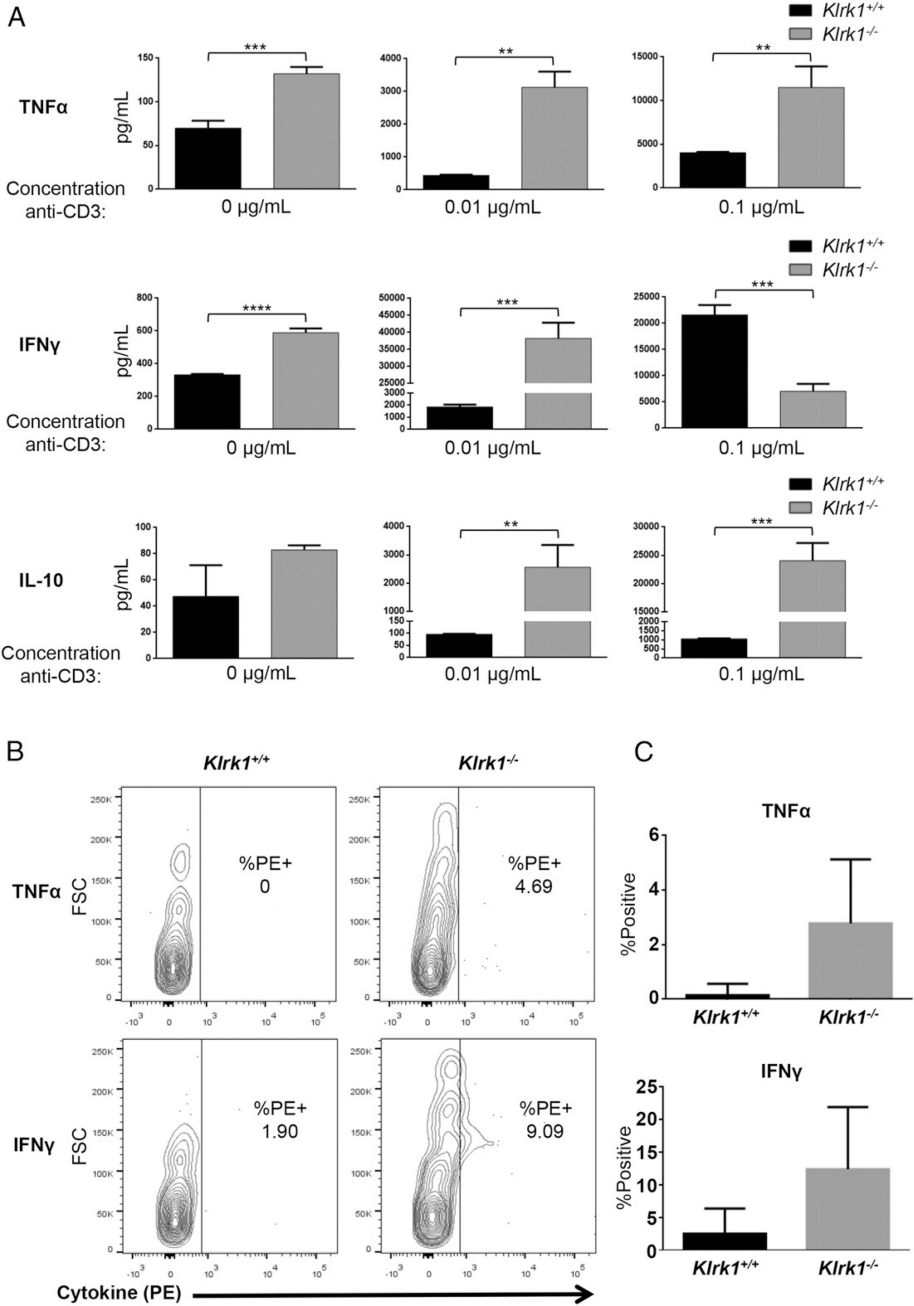
Altered cytokine production by *Klrk1*^−/−^ NOD CTL (**A**) Secretion of TNF-α, IFN-γ, and IL-10 (mean ± STD) by in vitro–generated *Klrk1*^−/−^ and wild-type CTL stimulated with the indicated concentrations of anti-CD3ε Ab. Data are representative of at least six independent experiments. (**B**) Representative flow cytometry plots showing intracellular staining of TNF-α and IFN-γ in *Klrk1*^−/−^ and wild-type NOD CD8^+^ T cells 1 wk after cotransfer into a wild-type NOD adoptive transfer recipient mouse. (**C**) Combined results (mean ± SEM) from transfers into eight mice from three independent experiments showing the percent TNF-α or IFN-γ positive *Klrk1*^−/−^ and wild-type NOD CD8^+^ T cells 1 wk after cotransfer into wild-type NOD adoptive transfer recipient mice. **p ≤ 0.01, ***p ≤ 0.001, ****p ≤ 0.0001 in two-tailed unpaired Mann–Whitney *U* test.

In the absence of stimulation, or with a low concentration of anti-CD3 Ab, *Klrk1*^−/−^ CTL produced significantly more IFN-γ, TNF-α, and IL-10 compared with wild-type CTL (Fig. 4A, Supplemental Fig. 1A). With a higher concentration of anti-CD3 Ab, *Klrk1*^−/−^ CTL also produced greater amounts of TNF-α and IL-10 (Fig. 4A, Supplemental Fig. 1A). At this higher level of stimulation, IFN-γ production by *Klrk1*^−/−^ CTL was lower in some experiments compared with wild-type CTL; however, this did not reach statistical significance when data from multiple experiments were combined (Fig. 4A, Supplemental Fig. 1A). In contrast to this effect on cytokine production, no change in lytic granule release was observed (Supplemental Fig. 1B). To determine if NKG2D expression similarly affected NOD CD8^+^ T cell cytokine production in vivo, we purified CD8^+^ T cells from *Klrk1*^−/−^ or wild-type NOD mice and labeled them with eFluor 670 or CFSE, respectively. Without providing exogenous TCR activation, we adoptively transferred a 1:1 mixture of these cells into wild-type NOD recipient mice (Supplemental Fig. 2A). After 1 wk, *Klrk1*^−/−^ cells recovered from the spleen produced more TNF-α and IFN-γ than wild-type cells recovered from the same spleen (Fig. 4B, 4C). Together, these results demonstrate that expression of NKG2D by NOD CD8^+^ T cells generally reduces effector cytokine production by these cells.

### NKG2D–H60a interaction during NOD CTL differentiation dampens diabetogenic cytokine production

**FIGURE 5:**
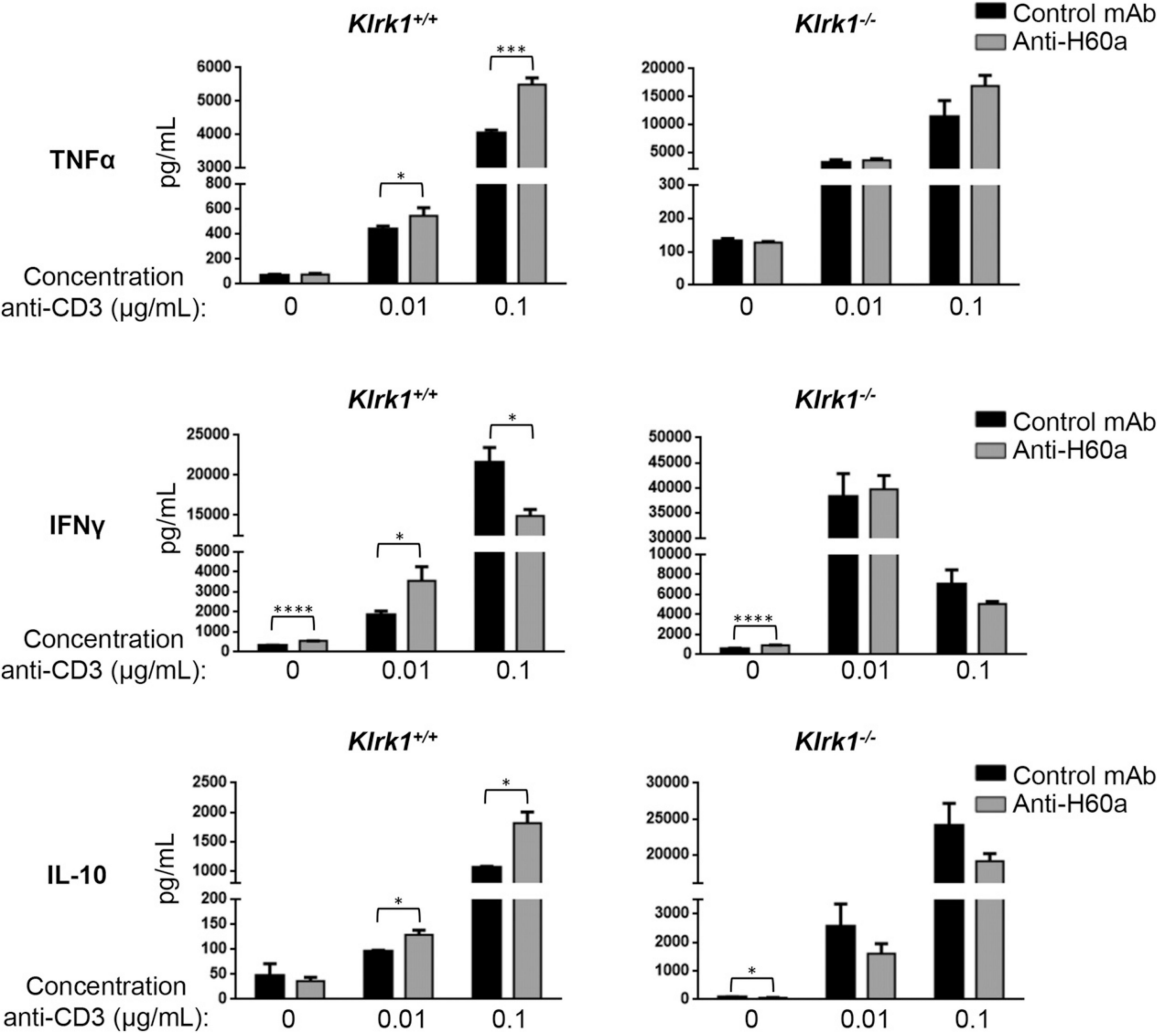
Blockade of NKG2D–H60a interaction during NOD CTL differentiation results in increased CTL effector cytokine production Secretion of TNF-α, IFN-γ, and IL-10 by in vitro–generated *Klrk1*^−/−^ and wild-type CTL stimulated with the indicated concentrations of anti-CD3ε Ab in the presence of an anti-H60a or isotype control Ab. Data are representative of at least six independent experiments. *p ≤ 0.05, ***p ≤ 0.001, ****p ≤ 0.0001 in two-tailed unpaired Mann–Whitney *U* test.

To assess whether the increased cytokine production by *Klrk1*^−/−^ CTL was the result of a loss of NKG2D interaction with H60a during the effector response, we included a blocking Ab against H60a during effector CTL stimulation. However, this blockade did not affect cytokine secretion by any of the CTLs (Supplemental Fig. 3A, 3B). This led us to hypothesize that NKG2D–H60a interaction during CTL differentiation, rather than during the effector CTL response, was responsible for the altered cytokine production by *Klrk1*^−/−^ CTL. To test this, we added anti-H60a blocking Ab at the beginning of the T cell culture. This resulted in increased TNF-α, IL-10, and IFN-γ by wild-type CTL when the cells were stimulated with a low concentration of anti-CD3 Ab (Fig. 5, Supplemental Fig. 4). With a higher concentration of anti-CD3 Ab, H60a blockade also resulted in greater production of TNF-α and IL-10, but lower or similar production of IFN-γ, by wild-type CTLs (Fig. 5, Supplemental Fig. 4). Confirming this effect of H60a blockade was dependent on NKG2D expression, no difference in cytokine production was observed between CD3-stimulated anti–H60a-and isotype control-treated *Klrk1*^−/−^ CTL (Fig. 5, Supplemental Fig. 4). The similar effect of NKG2D deficiency (Fig. 4A, Supplemental Fig. 1A) and H60a blockade during CTL differentiation (Fig. 5, Supplemental Fig. 4) demonstrates that NKG2D–H60a interaction via homotypic CD8^+^ T cell contact during CTL differentiation alters the NOD CTL effector cytokine response, generally reducing production.

### NKG2D enhances diabetes development in SPF-housed NOD mice

**FIGURE 6:**
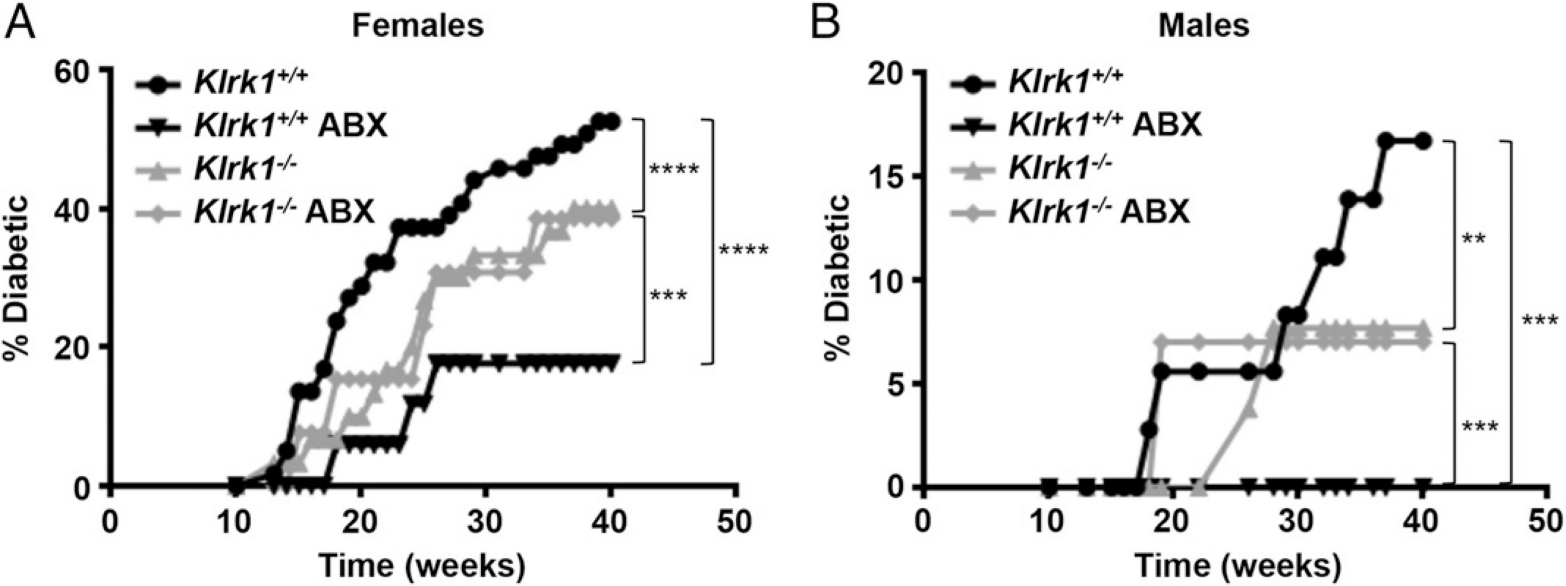
NKG2D expression affects NOD diabetes development and is protective in microbiota-depleted NOD mice Cumulative diabetes incidence in (**A**) female wild-type (*n* = 59), antibiotic (ABX)-treated wild-type (*n* = 17), *Klrk1*^−/−^ (*n* = 30), ABX-treated *Klrk1*^−/−^ (*n* = 13) and (**B**) male wild-type (*n* = 36), ABX-treated wild-type (*n* = 10), and *Klrk1*^−/−^ (*n* = 26) and ABX-treated *Klrk1*^−/−^ (*n* = 14) NOD mice. **p ≤ 0.002, ***p ≤ 0.001, ****p ≤ 0.0001 in two-way ANOVA.

Expression of TNF-α, IFN-γ, and IL-10 within the islets all enhance NOD insulitis and diabetes development (37). Given the increased production of these cytokines by *Klrk1*^−/−^ CTL, we predicted that NKG2D expression would have a protective role in NOD diabetes development. To test this, we first compared diabetes development between *Klrk1*^−/−^ and wild-type NOD littermates housed under SPF conditions. As is well characterized in the NOD model (38–40), there was greater diabetes development in the female compared with the male mice. However, in both sexes, the *Klrk1*^−/−^ mice exhibited slower diabetes development compared with wild-type mice (Fig. 6).

### NKG2D decreases diabetes in NOD mice treated with broad-spectrum antibiotics

The reduced diabetes development in *Klrk1*^−/−^ NOD mice was inconsistent with our finding that NKG2D–H60a interaction decreases CTL effector function. This suggested there is an additional prodiabetic role for NKG2D signaling in disease development. We hypothesized that this role may be via interaction with the microbiota. This was based on the fact that *Klrk1*^−/−^ mice are deficient in B1a B cells (41), which are believed to play a major role in shaping the microbiota composition via IgA production (24), and that NOD diabetes development is sensitive to microbiota alterations (40, 42–44). To test this hypothesis, we compared the effect on diabetes development of an antibiotic treatment that largely depletes the intestinal microbiota (45–47). Compared with SPF-housed wild-type mice, antibiotic-treated wild-type NOD mice exhibited reduced diabetes development (Fig. 6). By contrast, diabetes development in *Klrk1*^−/−^ mice was unchanged by antibiotic treatment (Fig. 6). These results demonstrate that an interaction exists between NKG2D and the microbiota present in our SPF mouse colony, which enhances NOD diabetes incidence. By contrast, with antibiotic treatment, diabetes development was enhanced in *Klrk1*^−/−^ compared with wild-type NOD mice (Fig. 6). Similar to SPF-housed mice, adoptively transferred *Klrk1*^−/−^ cells recovered from the spleen of antibiotic-treated wild-type mice produced more TNF-α and IFN-γ compared with adoptively transferred wild-type cells (Supplemental Fig. 2B). These results support our findings that NKG2D plays a protective role during NOD diabetes development via interaction with H60a during CTL differentiation.

**FIGURE 7:**
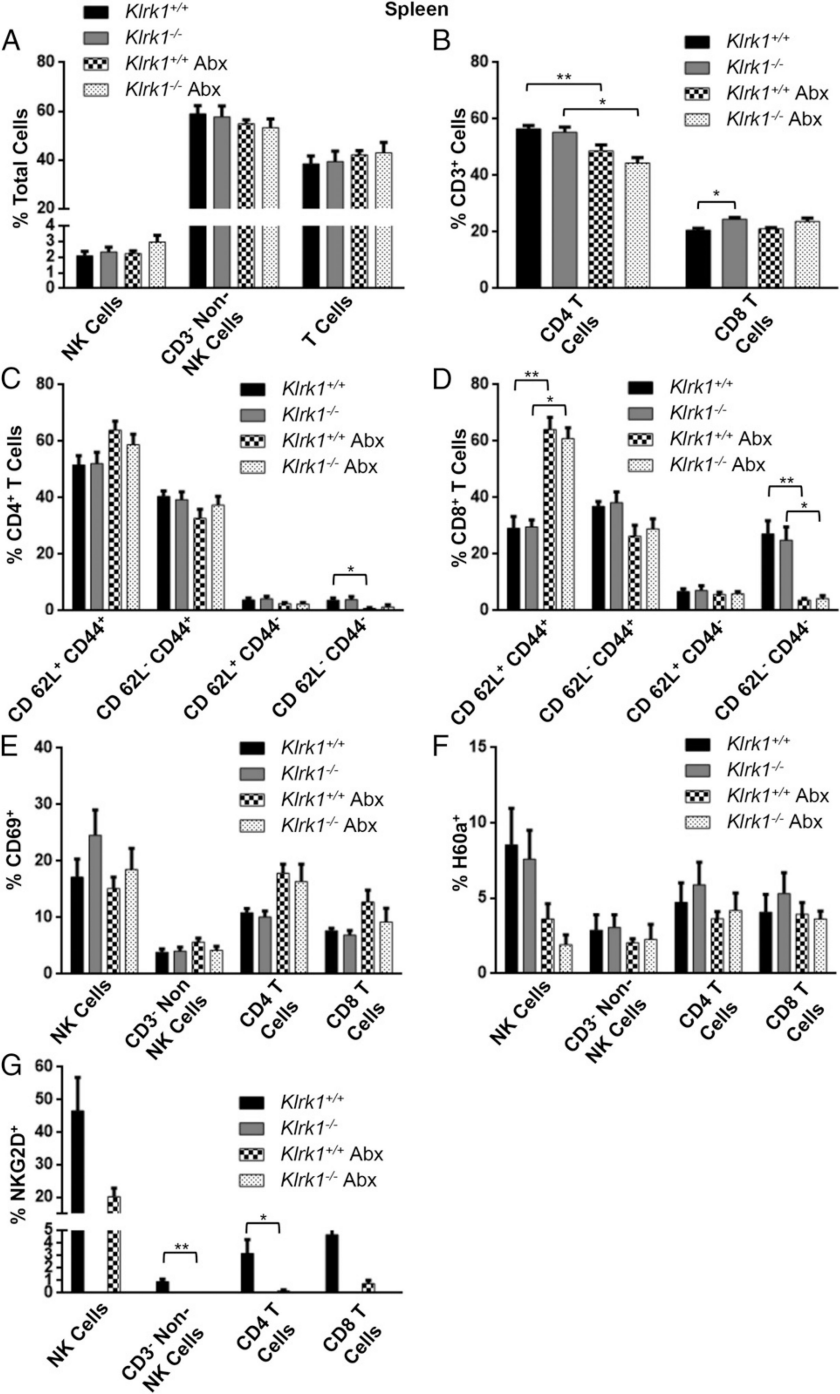
Immune cell populations, activation, and NKG2D and H60a expression in the spleen of untreated and antibiotic-treated *Klrk1^+/+^* and *Klrk1*^−/−^ NOD mice (**A**) Percentage of total splenocytes in 12 wk old wild-type and *Klrk1*^−/−^ NOD mice with or without antibiotic that were CD3^−^ CD49b^+^ (NK cells), CD3^−^ CD49b^−^ (non-T or NK cells), or CD3^+^ (T cells). (**B**) Percentage of splenic T cells in (A) that were CD4^+^ and CD8^+^. (**C**) CD62L and CD44 expression by splenic CD4^+^ T cells in (A). (**D**) CD62L and CD44 expression by splenic CD8^+^ T cells in (A). (**E**–**G**) Percentage of splenic populations in (A) and (B) that expressed (E) CD69, (F) H60a, or (G) NKG2D. Data are combined results (mean ± SEM) of at least four mice per group. *p ≤ 0.05, **p ≤ 0.01 in two-tailed unpaired Mann–Whitney *U* test.

**FIGURE 8:**
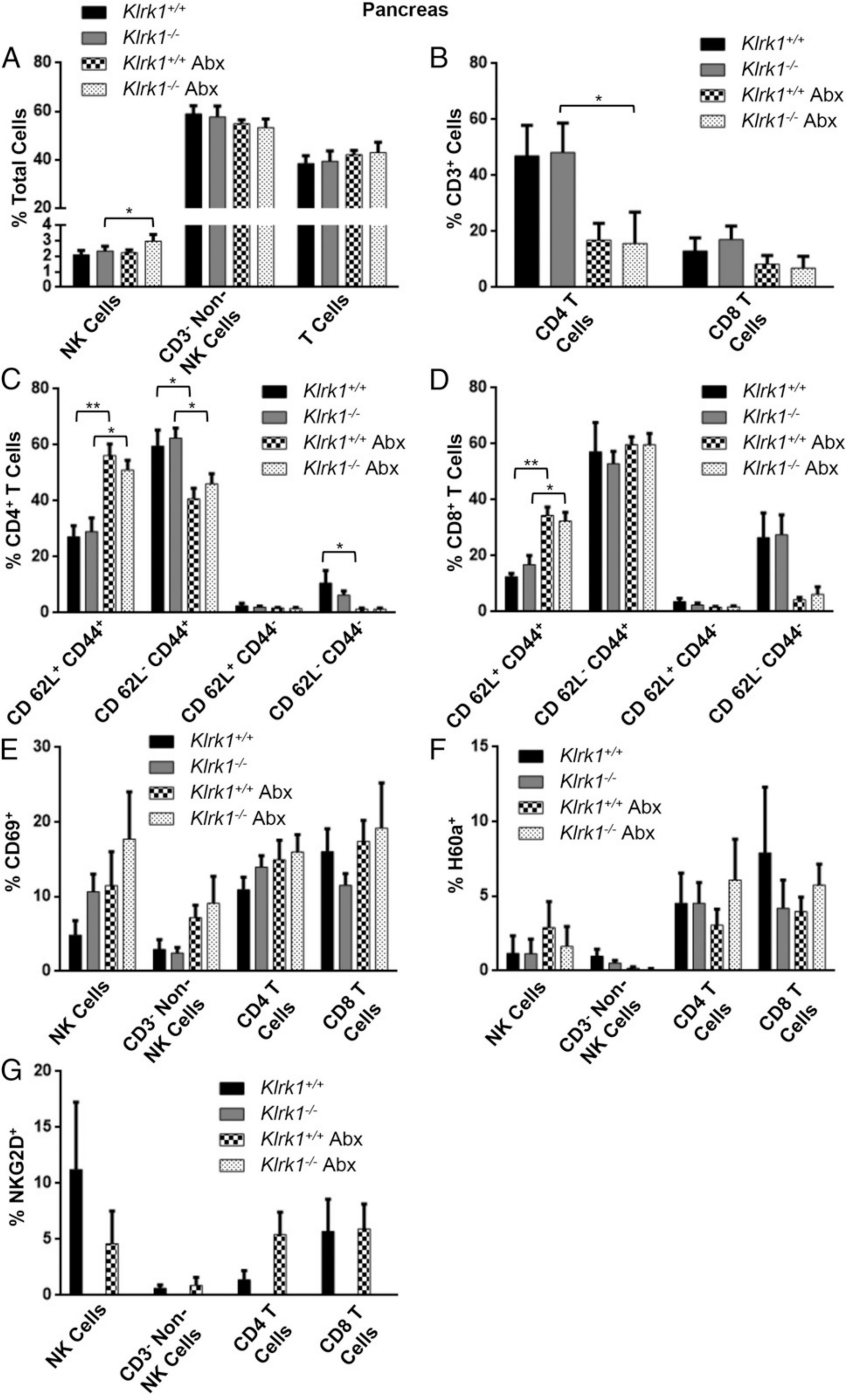
Immune cell populations, activation, and NKG2D and H60a expression in the pancreata of untreated and antibiotic-treated *Klrk1^+/+^* and *Klrk1*^−/−^ NOD mice (**A**) Percentage of cells isolated from the pancreata of 12 wk old wild-type and *Klrk1*^−/−^ NOD mice with or without antibiotic that were CD3^−^ CD49b^+^ (NK cells), CD3^−^ CD49b^−^ (non-T or NK cells), or CD3^+^ (T cells). (**B**) Percentage of T cells in (A) that were CD4^+^ and CD8^+^. (**C**) CD62L and CD44 expression by CD4^+^ T cells in (A). (**D**) CD62L and CD44 expression by CD8^+^ T cells in (A). (**E**–**G**) Percentage of populations in (A) and (B) that expressed (E) CD69, (F) H60a, or (G) NKG2D. Data are combined results (mean ± SEM) of at least four mice per group. *p ≤ 0.05, **p ≤ 0.01 in two-tailed unpaired Mann–Whitney *U* test.

We next compared the immune cell populations present in the spleen and pancreas of wild-type and *Klrk1*^−/−^ mice under both SPF and antibiotic-treated conditions. Antibiotic treatment did not significantly change NKG2D or H60a expression (Figs. 7G, 8G), and there was no difference in CD69 expression on immune cells in the pancreas or spleen between wild-type and *Klrk1*^−/−^ mice under either condition (Figs. 7E, 8E). Antibiotic treatment did result in a decrease in CD4^+^ T cells in both the pancreas and spleen (Figs. 7B, 8B), and altered the ratio of effector to central memory CD4^+^ T cells in the pancreas (Fig. 8C). Specifically, with antibiotics there were fewer effector CD4^+^ T cells (CD44^+^CD62L^−^) and more central memory CD4^+^ T cells (CD44^+^CD62L^+^) in the pancreas in both wild-type and *Klrk1*^−/−^ mice (Fig. 8C). There was a similar increase in central memory CD8^+^ T cells in the pancreas; however, unlike the CD4^+^ T cell population, there was no corresponding decrease in effector CD8^+^ T cells (Fig. 8D). These data demonstrate that there were fewer effector T cells in the pancreas of the antibiotic-treated mice. This corresponds with the reduced diabetes development in these mice compared with SPF-housed wild-type mice (Fig. 6), as well as the increased ability to observe the protective role of NKG2D in antibiotic-treated mice (Fig. 6).

## DISCUSSION

In this study we demonstrate that NKG2D expression affects NOD diabetes development via two previously undescribed mechanisms. First, we demonstrate that interaction between NKG2D and H60a, both expressed on differentiating CTL, reduces the production of effector cytokines by NOD CTL. This corresponds with decreased diabetes development in antibiotic-treated *Klrk1*^−/−^ NOD mice. Second, we showed that NKG2D influences NOD diabetes development via interaction with the microbiota.

NKG2D is implicated in the development of autoimmune diabetes in both humans and mice (7–10); however, a definitive role for NKG2D in this disease has not been established. Much of this uncertainty has been due to seemingly conflicting results in the NOD mouse model. Previous reports have demonstrated that NKG2D either promotes NOD diabetes (7) or has little effect (34). In particular, Ogasawara et al. (7) suggested that expression of the NKG2D ligand RAE1 in pancreatic islets targets islet β cells for NKG2D-mediated destruction. Our later finding that transgenic expression of RAE1 by islet β cells drove immune cell infiltration of pancreatic islets in the non-autoimmune C57BL/6 background seemed to support the feasibility of this model (8). In the current study, however, we did not detect expression of RAE1 mRNA, or any other NKG2D ligand, by CD45^−^ cells in pancreatic islets. This finding is consistent with other reports that found no RAE1 mRNA (32, 33), or protein (33) expressed by NOD islet cells. These differing reports of RAE1 expression may be a result of variance between NOD mouse colonies due to gut microbial influence or genetic drift (40, 42, 48). A report by Adlercreutz et al. (49) did show expression of H60a mRNA in the pancreatic islets of 13 wk old NOD and BALB/c mice, but did not distinguish between immune and nonimmune cells. This is consistent with the H60a mRNA expression observed in our study.

Our observation of robust H60a expression by activated NOD CD8^+^ T cells was not anticipated, but is consistent with earlier reports. H60a mRNA was previously shown to be expressed in healthy NOD tissues and by NOD NK cells (49, 50). Similar to the NOD mouse, BALB/c immune cells express H60a (23, 25, 51), and mRNA encoding this NKG2D ligand was shown to be upregulated in BALB/c T cells upon activation (52). Likewise, human T cells also express NKG2D ligands upon activation (53–55).

Constitutive NKG2D ligand expression decreases NKG2D surface expression on NK and CD8^+^ T cells (18, 56, 57). Poor NKG2D expression has been previously reported on NOD NK cells (50), which correlated with RAE1 expression by these cells (50). Similarly, the high level of H60a expression on NOD CD8^+^ T cells is likely responsible for the low surface expression of NKG2D we observed on these cells. However, our results clearly show that this low NKG2D expression has functional relevance to CTL differentiation.

To date, the function of NKG2D in CD8^+^ T cells has largely been studied in differentiated CTLs. These studies have generally shown a costimulatory role for NKG2D, enhancing TCR-stimulated effector function (11, 28, 58) or memory formation (59, 60). The effects of NKG2D signaling in the absence of TCR engagement are not well understood, but have been shown to induce immune synapse formation by mouse CTLs (28) and MHC-unrestricted killing by cytokine-activated human CTLs (61). Therefore, our finding that there is a role for NKG2D–H60a interaction during CD8^+^ T cell differentiation into CTL describes a new mode of NKG2D–ligand interaction, which dampens, rather than enhances, later CTL responses.

In our in vitro experiments, there was a consistent decrease in TNF-α, IL-10, and IFN-γ production by CTL with either genetic knockout of NKG2D or Ab blockade of H60a with low levels of stimulation. At the highest level of stimulation, IFN-γ production was significantly decreased in a number of experiments (Figs. 4, 5). This decrease could potentially reflect a different cytokine response at very high levels of TCR stimulation. However, because this decrease was not significant when data from all experiments were combined (Supplemental Figs. 1, 3), we did not investigate this further. Additionally, the consistently enhanced production of IFN-γ by *Klrk1*^−/−^ adoptively transferred CD8^+^ T cells in vivo (Fig. 4B, 4C) suggests that low to medium levels of TCR stimulation more closely reflect in vivo conditions.

When we assessed diabetes incidence in *Klrk1*^−/−^ and wild-type NOD mice, our results indicated a different effect of NKG2D genotype on diabetes incidence depending upon whether the mice were treated with microbiota-depleting antibiotics. Under SPF housing conditions, we found a detrimental role for NKG2D in NOD diabetes development. By contrast, when animals were treated with antibiotics, wild-type NOD mice had delayed disease compared with *Klrk1*^−/−^ mice. This differential effect revealed a separate protective role for NKG2D. In addition, the lack of an effect of antibiotic treatment on disease incidence in *Klrk1*^−/−^ mice provides evidence for NKG2D genotype–dependent changes in the microbiota affecting NOD diabetes incidence. Antibiotic-treatment resulted in a decrease in effector T cells in the pancreas. This is a probable mechanism by which diabetes development was decreased in antibiotic-treated compared with SPF-housed wild-type mice. Further, it is likely that this decrease in effector T cells is responsible for the increased ability to observe the protective role of NKG2D in diabetes development with antibiotic treatment. Taken together, our results lead us to propose a detrimental influence of NKG2D on diabetes development in the gut, which involves the microbiota, and a separate effect independent of microbiota in which NKG2D dampens the NOD CTL cytokine response.

We observed the well-established sexual dimorphism in NOD diabetes incidence, with increased rates of diabetes in females compared with males (38–40). Within SPF-housed or antibiotic-treated groups, however, the effect of NKG2D was consistent between males and females. The mechanism by which NKG2D promotes NOD diabetes through interactions with microbiota was not explored in the current study, but warrants further investigation. This may be due to a lack of B1a cells in these mice (41), or interaction of other immune cells with NKG2D ligands, which are constitutively expressed by intestinal epithelial cells (62–65). Additionally, further work remains to conclusively attribute the protective effect of NKG2D genotype in antibiotic-treated animals to the effects of NKG2D–H60a interactions between CD8^+^ T cells. Although our data do support this hypothesis, a definitive answer awaits the generation of a NOD H60a^−/−^ mouse, as well as CD8^+^ T cell conditional NOD NKG2D^−/−^ and NOD H60a^−/−^ animals. Finally, our model of opposing pro- and antidiabetic effects of NKG2D suggests that a lack of consensus from previous studies concerning the role of NKG2D in NOD diabetes may be the result of variation in cohousing practices and differences in microbiota between mouse colonies.

In summary, we found that NKG2D–H60a interaction between CD8^+^ T cells during NOD CTL differentiation in vitro resulted in decreased production of diabetogenic cytokines upon subsequent CTL activation. To our knowledge, this is the first report of a role for NKG2D–NKG2D ligand interaction during differentiation of mouse CD8^+^ T cells into CTL rather than during the effector phase of differentiated CTL. Furthermore, these results describe a functional role for NKG2D ligand expression by healthy murine CD8^+^ T cells. Finding that NKG2D is protective in animals with depleted microbiota correlates with NKG2D signaling resulting in reduced cytokine production by NOD CTL. The additional observation that microbiota-depleting antibiotics profoundly affected NOD diabetes development in wild-type, but not *Klrk1*^−/−^, animals demonstrates a separate effect in which NKG2D-dependent alterations in the microbiota promote disease development. Additionally, these data may have broader implications for the role of NKG2D–ligand interaction between T cells in modulating CD8^+^ T cell responses in both mice and humans.

## Supplementary Material



## Abbreviations used in this article

MICA: MHC class I chain–related A
NKG2D: NK group 2 member D
SPF: specific pathogen-free.
