# Supplementary material for: A Protective Role for NKG2D–H60a Interaction via Homotypic T Cell Contact in Nonobese Diabetic Autoimmune Diabetes Pathogenesis
Source: Immunohorizons. Author manuscript; Available in PMC 2018 Feb 27. (PMC5828234; doi:10.4049/immunohorizons.1700011)
Supplement: Supplementary file 1 [file NIHMS919956-supplement-supplement_1.pdf]

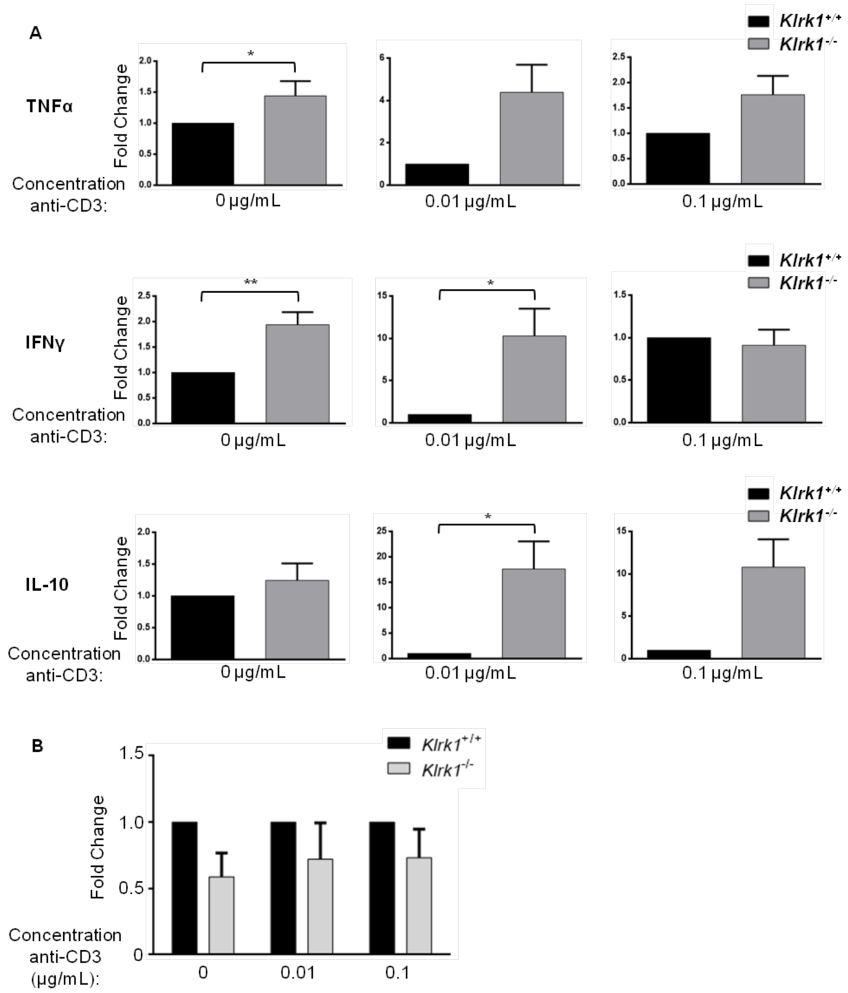

Figure S1. NKG2D deficiency alters NOD CTL cytokine production but not lytic granule release. (A) Fold change (mean +/- SEM) in TNFα, IFNγ and IL-10 secretion by *Klrk1*$^{-/-}$ CTL compared with wild type CTL. **(B)** Fold change (mean +/- SEM) in percent of CD107a+ *Klrk1*$^{-/-}$ CTL compared with percent of CD107a+ wild type CTL. Data are combined from at least 4 independent experiments. *p ≤ 0.05, **p ≤ 0.01, ***p ≤ 0.001, ****p ≤ 0.0001 in two-tailed Wilcoxon test.

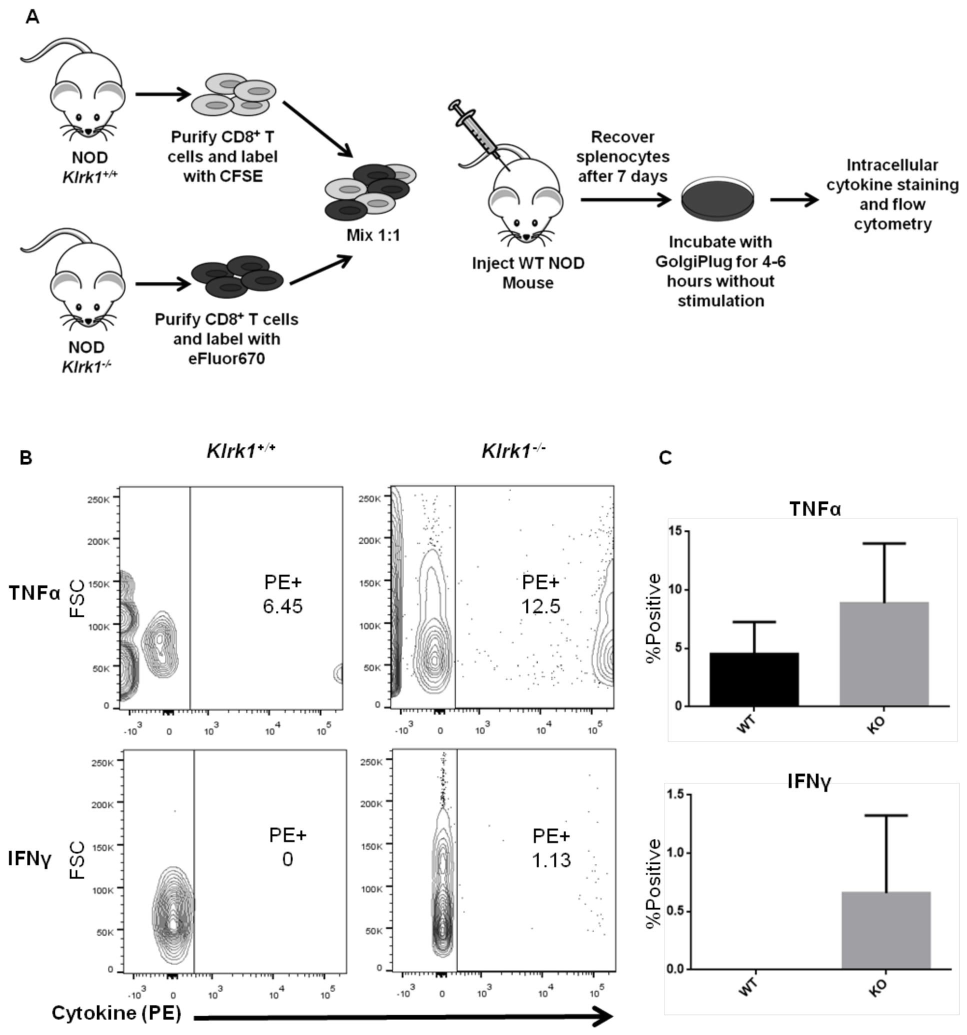

Figure S2. ***Klrk1***$^{-/-}$ **NOD CTL produce increased TNFα and IFNγ in antibiotic-treated mice.** **(A)** Schematic of adoptive transfer and intracellular cytokine staining of labeled CD8^{+} T cells from wild type and *Klrk1*$^{-/-}$ mice**.** **(B)** Representative flow cytometry plots showing intracellular staining for TNFα and IFNγ in *Klrk1*$^{-/-}$ and wild type NOD CD8^{+} T cells one week after co-transfer into wild type ABX-treated NOD adoptive transfer recipient mice. **(C)** Combined results of two independent experiments (mean+/-SEM) showing percent TNFα or IFNγ positive *Klrk1*$^{-/-}$ and wild type NOD CD8^{+} T cells 1 week after co-transfer into wild type ABX-treated NOD adoptive transfer recipient mice.

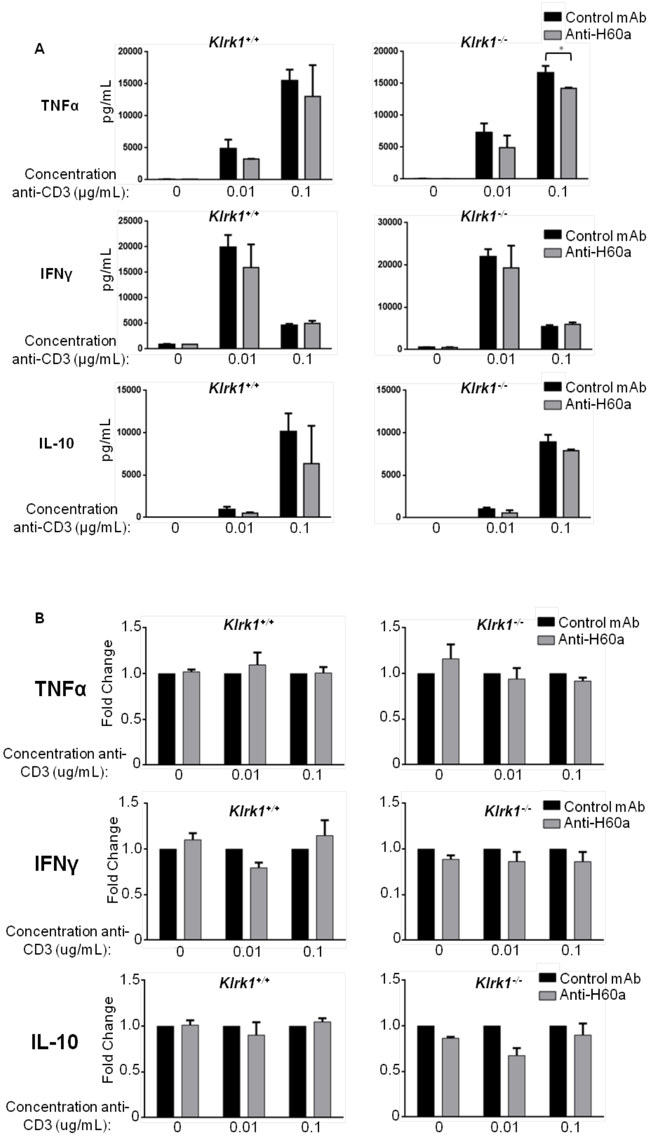

Figure S3. CTL effector cytokine production is not affected by blockade of NKG2D-H60a interaction during NOD CTL effector response. (A) Representative experiment showing TNFα, IFNγ and IL-10 secretion (mean +/- STD) by *Klrk1*$^{-/-}$ and wild type CTL stimulated with anti-CD3ε in the presence of an anti-H60a or isotype control antibody. Data are representative of at least 4 independent experiments. $*p \leq 0.05$ in one-tailed unpaired Mann-Whitney test. **(B)** Fold change (mean +/- SEM) in TNFα, IFNγ and IL-10 secretion by CTL stimulated with anti-CD3ε in the presence of anti-H60a compared with isotype control antibody. Data are combined from at least 4 independent experiments.

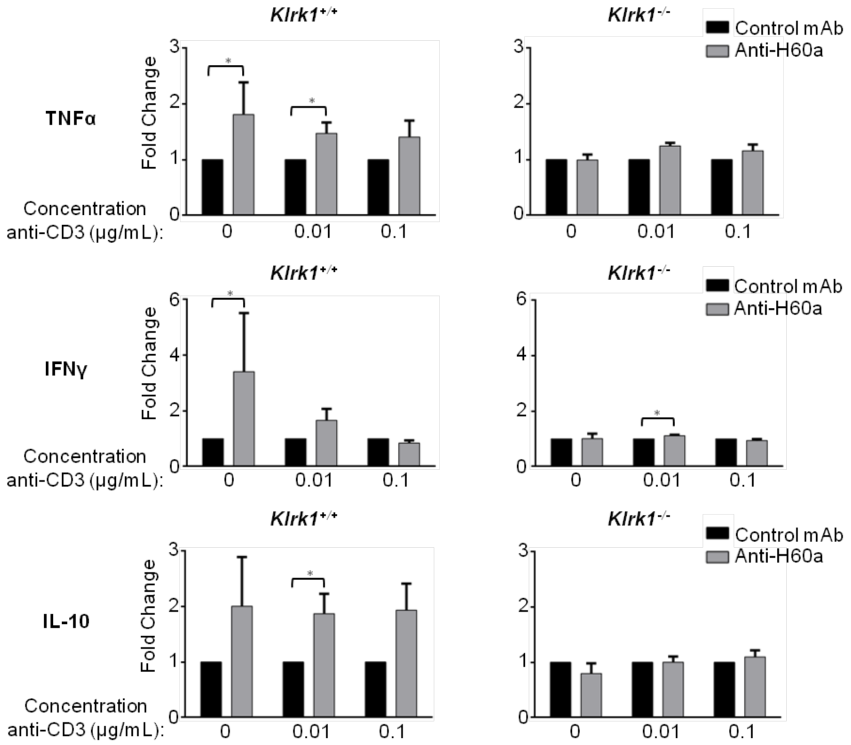

Figure S4. Blockade of NKG2D-H60a interaction during NOD CTL differentiation increases CTL effector cytokine production. Fold change (mean+/-SEM) in TNFα, IFNγ and IL-10 secretion by *Klrk1*$^{-/-}$ and wild type CTL stimulated with anti-CD3ε generated in the presence of an H60a blocking compared with isotype control antibody. Data are combined from at least 6 independent experiments. *$p \leq 0.05$ in one-tailed Wilcoxon test.
